# Physical Activity across Frailty Phenotypes in Females with Parkinson's Disease

**DOI:** 10.1155/2012/468156

**Published:** 2012-08-07

**Authors:** Kaitlyn P. Roland, Kayla M. D. Cornett, Olga Theou, Jennifer M. Jakobi, Gareth R. Jones

**Affiliations:** ^1^Interdisciplinary Graduate Studies, Health and Exercise Sciences, University of British Columbia Okanagan, Kelowna, BC, Canada V1V 1V7; ^2^Faculty of Geriatrics Medicine, Dalhousie University, Halifax, NS, Canada B3H 2E1; ^3^School of Health and Exercise Sciences, Faculty of Health and Social Development, University of British Columbia Okanagan, 3333 University Way, Kelowna, BC, Canada V1V 1V7

## Abstract

Females with Parkinson's disease (PD) are vulnerable to frailty. PD eventually leads to decreased physical activity, an indicator of frailty. We speculate PD results in frailty through reduced physical activity. *Objective*. Determine the contribution of physical activity on frailty in PD (*n* = 15, 65 ± 9 years) and non-PD (*n* = 15, 73 ± 14 years) females. *Methods*. Frailty phenotype (nonfrail/prefrail/frail) was categorized and 8 hours of physical activity was measured using accelerometer, global positioning system, and self-report. Two-way ANCOVA (age as covariate) was used to compare physical activity between disease and frailty phenotypes. Spearman correlation assessed relationships, and linear regression determined associations with frailty. *Results*. Nonfrail recorded more physical activity (intensity, counts, self-report) compared with frail. Self-reported physical activity was greater in PD than non-PD. In non-PD, step counts, light physical activity time, sedentary time, and self-reported physical activity were related to frailty (*R* = 0.91). In PD, only carbidopa-levodopa dose was related to frailty (*r* = 0.61). *Conclusion*. Physical activity influences frailty in females without PD. In PD females, disease management may be a better indicator of frailty than physical activity. Further investigation into how PD associated factors contribute to frailty is warranted.

## 1. Introduction

Frailty is a geriatric syndrome that results in an increased vulnerability to acute and chronic illness, falls and related injuries, and a general loss of functional independence [1–4]. The cardiovascular health study frailty index (CHS*fi*), proposes that frailty develops across a spectrum of phenotypes ranging from nonfrail to prefrail to frail [5]. All stages of frailty are evident within community-dwelling populations, with over 70% of older adults expressing some frailty characteristics [6]. Frailty is twice as prevalent in females as males and females typically display more frailty characteristics than males [5, 7]. Females experience increased frailty severity than males since they live longer and spend a greater proportion of life managing disability and disease [7–9]. 

Females with Parkinson's disease (PD) are at an increased risk of frailty [10], yet the presence of frailty in persons with PD may be misinterpreted as disease-related functional decline [11]. Females with PD are typically older [12], cite greater disability [13] and experience more difficulty performing activities of daily living (ADL) than males with PD [14, 15]. Recent evidence suggests that of the five CHS*fi* criteria, self-reported exhaustion best determines frailty phenotype in females with PD [10]. Exhaustion is a common complaint among older females, especially those with PD, and is strongly associated with inhibiting physical activity and ADL participation [16–18]. The established relationship between reduced physical activity and frailty severity [19] leads us to speculate that PD influences frailty through decreased participation in physical activity. Understanding how physical activity contributes to frailty in females with PD is important in directing management strategies aimed at maintaining functional independence. The objective of this study was to determine the contribution of daily physical activity on frailty phenotype in community-dwelling females with PD, compared to non-PD females.

## 2. Methods

Females greater than 50 years of age, living independently in the community, with mild to moderate PD severity (Hoehn & Yahr stage 1–3) were recruited through advertisements and support group presentations. Non-PD females were similarly recruited from the same local region. All participants were able to ambulate independently (with or without walk-aid). Females with PD were in a steady clinical state (controlled by medication) and cognitively intact. Females with PD were instructed to continue daily dopamine medication as prescribed and no incidence of freezing of gait or severe dyskinesia episodes were reported during the testing day. All participants provided written informed consent. The Clinical Research Ethics Board of the University of British Columbia granted ethical approval for this investigation. 

Health history questionnaires, physical frailty criteria (CHS*fi*), self-reported physical activity, and setup of daily physical activity monitors (accelerometer, GPS) were completed at the participant's home in the morning (8~10 am). All PD participants were assessed between 1 and 2 hours post anti-Parkinson's medication, and controls were assessed 1 hour after breakfast. In older adults, reduction in daily physical activity, and associated physiological change, can be quantified using continuous objective physical activity monitors (i.e., accelerometers and global positioning systems, GPS) [20]. In this study, a waist-borne accelerometer and wrist-born GPS recorded physical activity; and the participant wore these devices continuously for the entire testing day. The participant was then instructed to go about their typical daily activities, which they recorded in a written hourly log. The accelerometer, GPS, and physical activity logbook were collected approximately 7 hours later (between 4–7 pm) at the participant's home.

### 2.1. Frailty Phenotype

Frailty was categorized according to CHS*fi *[5] that includes five select criteria to determine a frailty phenotype (nonfrail, prefrail, and frail). These criteria include: (1) Unintended weight loss (>10 lbs in past 12 months); (2) Weakness (maximal handgrip strength classified by body mass index, BMI); (3) Walk speed (15 ft at usual pace classified by height); (4) Self-reported energy expenditure (Minnesota Leisure Time Activities Questionnaire, MLTA); (5) Self-reported exhaustion (Center for Epidemiological Studies depression scale, CES-D). Participants were considered nonfrail if they satisfied none of the phenotypic criteria, prefrail if they satisfied 1 or 2 criteria, and frail if they satisfied 3 or more criteria [5].

### 2.2. Accelerometer

Daily physical activity was measured using an ActiTrainer accelerometer (Actigraph, LLC, Fort Walton Beach, FL) secured in a holster worn at the waist on the dominant side. The ActiTrainer (8.6 × 3.3 × 1.5 cm; 51 grams) is a triaxial solid-state accelerometer that was programmed to record 60-second epochs of data. Data was uploaded to ActiLife5 v.5.8.3 software (ActiGraph, LLC, Fort Walton Beach, FL). Physical activity intensity levels were categorized according to the cut-points described by Copeland and Eslinger for older adults [21]. Sedentary activity was defined as 0 to 50 counts per minute, light physical activity as 51 to 1,040 counts per minute, and moderate-to-vigorous physical activity as greater than 1,041 counts per minute. Percentage of time spent at each level of activity was reported. Measurement outputs included total counts (i.e., daily step and activity counts, total minutes of activity) and intensity (i.e., percentage of time spent at sedentary, light, and moderate-vigorous activity intensity levels).

### 2.3. Global Positioning System (GPS)

Global Positioning System (GPS) examines gross mobility outside the individuals' home. GPS used in combination with the accelerometer can accurately assess physical activity within real-life environments [22], and this can be applied to categorize stages of frailty [23]. Participants wore a Garmin Forerunner 405 GPS watch (Garmin International Inc., Olathe, KS). GPS data were uploaded to the Garmin training center software (http://connect.garmin.com/). Both the GPS and accelerometer units were synchronized to record minute-by-minute data. The GPS and accelerometer data were time-matched using the ActiGraph GPS Correlation Wizard v.1.0.0 (Actigraph, LLC, Fort Walton Beach, FL) and exported in a Microsoft Excel compatible format for analysis. Only physical activity completed outside the home, defined using Garmin Training Software and an accompanying Google Earth Map, was included in the analysis. Participants' hourly physical activity log was also compared to GPS outputs to confirm physical activity participation. Vehicle-generated activity was considered any recording that measured greater than 3 m/sec for >1 min. All activity at speeds less than 3 m/sec >1 min were included as participant physical activity. GPS measurement outputs included; total GPS distance (km, vehicle- and participant-generated) and total amount of physical activity time (min).

### 2.4. Self-Reported Physical Activities

Self perceived energy expenditure was assessed using the self-reported MLTA [24], which was also used to determine energy expenditure as part of the CHS*fi* frailty assessment [5]. Twenty activities were specified, including walking for exercise, moderately strenuous household chores, mowing the lawn, raking the lawn, gardening, hiking, jogging, biking, exercise cycling, dancing, aerobics, bowling, golfing, calisthenics/general exercise, swimming, doubles tennis, singles tennis, and racquetball. Participants who engaged in any physical activity during the previous 2-weeks recorded the number of sessions and their duration. Energy (kcal/week) expenditure was determined using metabolic equivalent (MET) score: (activity-specific MET) × ((activity duration in minutes)/60) × ((number of sessions in past two weeks)/2). Total energy expenditure was calculated by summing expenditures over all activities.

### 2.5. Statistical Analysis

All analysis was performed using Statistical Package for Social Science 18.0 (PASW Statistics 18.0, SPSS Inc. IBM Somers, NY). Subject characteristics were compared between groups (non-PD, PD) and between frailty phenotype (nonfrail, prefrail, and frail) with a one-way ANOVA. The non-PD females were older than PD (*P* = 0.005), nonfrail females were younger than prefrail and frail (*P* = 0.007), and prefrail were younger than frail (*P* = 0.03). Thus, age was used as a covariate in a two-way ANCOVA to assess differences in physical activity between disease states (PD; non-PD) and frailty phenotype (nonfrail; prefrail; frail). To evaluate the main effects a univariate analysis was performed for each dependent variable to identify contributions to the main effects of disease states and frailty phenotypes. Probability level was set at *P* < 0.05 and Tukey post hoc tests were used to probe statistical interactions. Spearman's rank correlation was used to evaluate physical activity measures relative to frailty phenotype in each group. The physical activity measures that were significantly correlated with frailty phenotype were entered into a multiple regression analysis model with frailty phenotype as the dependent variable and physical activity measures as the independent variables. 

## 3. Results

Fifteen persons with PD (65 ± 9 years) and 15 non-PD controls (73 ± 14 years) participated. Both groups were categorized into frailty phenotypes according to the CHS*fi *(Table 1). In females with PD, tremor in the upper limb was controlled with medication and participants self-reported no freezing of gait or periods of dyskinesia over the course of the day. Further, any reports of rigidity and/or slowness of movement (bradykinesia) were mild and did not restrict ADL. Comparison of physical activity between disease states and frailty phenotypes with age as a covariate determined nonfrail recorded less sedentary time, participated in more light-intensity physical activity and accumulated more steps compared with frail (Table 2). Also, self-reported physical activity was lower in frail compared with the nonfrail and prefrail phenotypes (Table 2). However, higher self-reported physical activity was reported in PD compared with non-PD, although the other physical activity variables did not differ between disease groups (Table 2).

### 3.1. Physical Activity and Frailty

**Figure 1:**
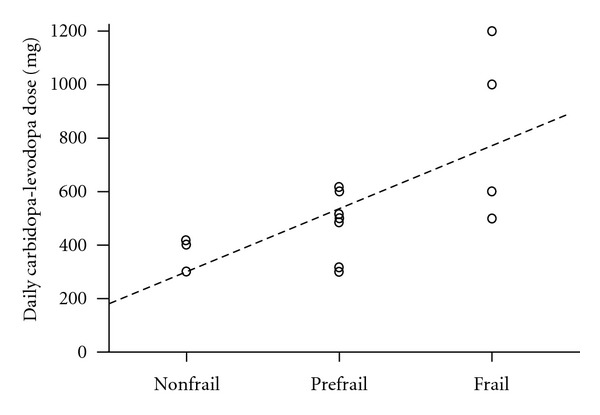
Relationship between frailty severity, measured by Cardiovascular Health Study frailty index, and daily carbidopa-levodopa dose (mg). Increased step count is positively correlated with greater frailty severity (*r* = 0.61) in females with PD. Females with PD are represented by the *open circles* and *dashed regression line*.

**Figure 2:**
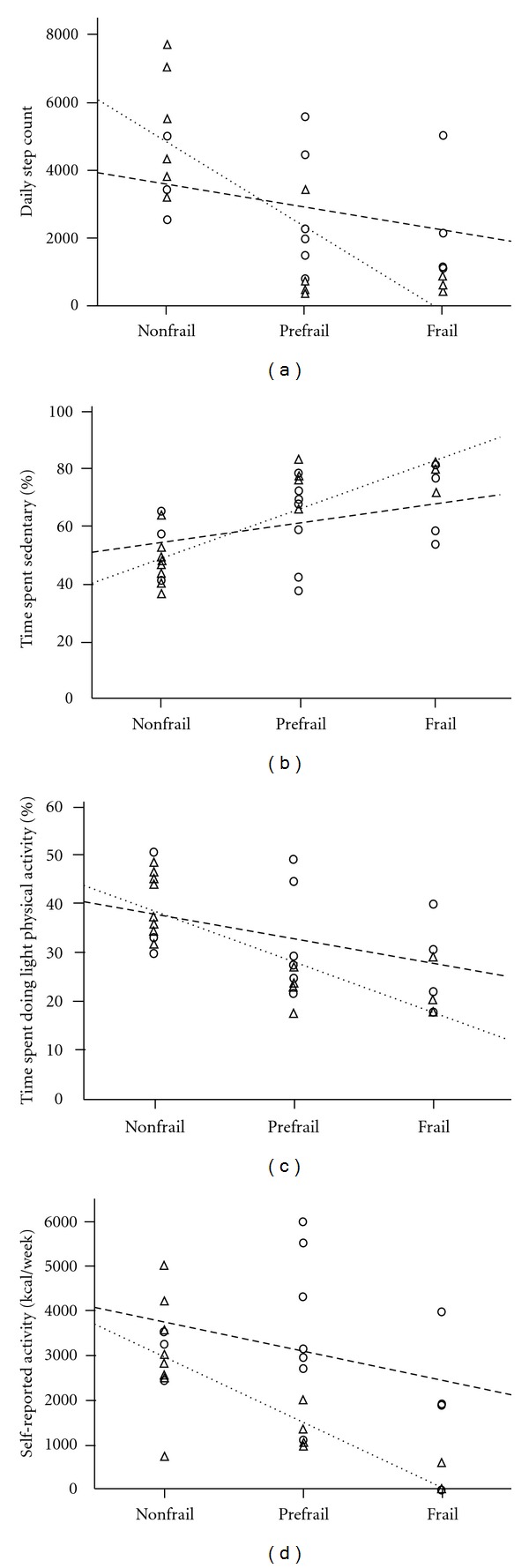
Relationship between frailty severity and: (a) daily step count. Total daily step count was negatively correlated with frailty severity (*r* = −0.79) in females without PD; (b) accumulated daily sedentary time. Sedentary time was positively correlated with frailty severity (*r* = 0.84) in females without PD; (c) accumulated light physical activity time. Light activity time was negatively correlated with frailty severity (*r* = −0.82) in females without PD; (d) self-reported leisure activity. Self-reported activity was negatively correlated with greater frailty severity (*r* = −0.82) in females without PD. Females with PD are represented by the *open circle* and *dashed regression line*; females without PD are represented by *the open triangle* and *dotted regression line*.

In PD, no physical activity variables were significantly related to frailty (*P* > 0.29); however, daily dose of carbidopa-levodopa, including both controlled-release and active-release forms, was correlated with frailty (*r* = 0.61; *P* = 0.01) (Figure 1). Other medication regimes for the management of PD and other comorbidities did not relate to frailty (*P* > 0.42). Physical activity variables demonstrated a significant linear relationship with increasing frailty severity in non-PD (Table 3). Those physical activity variables that were significantly different between frailty phenotypes (Table 3) were entered into individual regression models for non-PD. Low step, counts, higher sedentary behaviour, reduced light activity and lower weekly self-reported energy expenditure accounted for 83.3% of the variance (*R* = 0.913; *P* = 0.002; Figures 2(a)–2(d), but no single variable in this model best-determined frailty (*P* = 0.108 to 0.226). 

## 4. Discussion

This study examined the relationship between physical activity and frailty phenotype in community-dwelling females with PD and non-PD controls. Results of this study demonstrate physical activity (i.e., accelerometry counts and intensity, GPS, and self-reported) was not related to frailty phenotype in this sample group of females with PD; however, lower daily physical activity was associated with increased frailty severity in non-PD females. Current literature demonstrates physical inactivity as an important determinant of frailty phenotype [25], and persons with PD have reduced levels of accelerometry-assessed physical activity compared to non-PD controls [26]. This study suggests daily physical activity participation may not be the primary factor associated with frailty phenotype in females with PD. It is unclear from our cross-sectional data if a causal relationship exists between physical activity and frailty. However, results presented here support future longitudinal investigation into how PD progression impacts physical activity and how these changes in symptom expression and physical activity influence physical frailty.

### 4.1. Physical Activity: Not Related to Frailty Phenotype in PD

No relationship was demonstrated between daily physical activity and frailty phenotype in these females with PD. Although PD progression eventually debilitates motor performance, it is not likely to be the primary contributing factor to frailty in these females with PD. The results presented here do not necessarily exclude the contribution of physical activity to frailty, but rather suggest disease management may be a greater contributor to frailty. There is considerable variation in the manifestation of PD symptoms, which necessitates further large-scale investigation into PD-related contributors to frailty over the disease course. During the first 10 years of the disease, symptoms such as akinesia and festinating gait may not have progressed to the point at which they inhibit physical activity participation, which was the case with these participants [27]. In the initial stages of the disease, slowness to execute day-to-day activities (i.e., bradykinesia) is more common, and this likely contributes to perceived exhaustion, which is a criterion for frailty. Thus, in community-dwelling persons with PD, self-reported exhaustion resulting from PD symptoms contributes to frailty [10], rather than reduced daily physical activity. 

Results of this study indicate that although physical activity was not related to frailty in PD, females with PD self-reported greater leisure time activities compared to non-PD controls, regardless of frailty severity. This finding argues against previous research that states community-dwelling persons with PD have significantly less daily physical activity energy expenditure (measured using MLTA questionnaire) compared with controls, although the study sample included only males [28]. We speculate that increased self-reported leisure activity in these females with PD is due to the benefits of physical exercise being well recognized in the PD population to improve motor performance, functional and cognitive ability, safety, and confidence in ADL [27]. Persons living with PD are constantly encouraged to remain active despite disease-associated barriers (i.e., exhaustion) [29, 30]. Previous study demonstrated older adults with an increased risk of mortality, like persons with PD, adhere better to exercise programs compared to general community-dwelling older adults [31]. Therefore, we speculate that persons with PD may be involved in more physical activity and adhere better to exercise recommendations compared with community-dwelling counterparts because of PD-associated disease symptoms, regardless of frailty phenotype.

Unlike females with PD, physical activity influences frailty severity in community-dwelling females without PD. The importance of dedicating a greater percentage of daytime hours to light-intensity activities and decreasing sedentary time is highlighted in community-dwelling females. Sedentary time was related to increased frailty in females without PD, likely through its influence on physiological decline (i.e., decreased fitness), leading to greater dependence [32]. The contribution of physical activity to frailty in females without PD may be attributed to reduced mobility, less activity intensity and restricted life-space (i.e., the spatial area a person moves through during daily life) [33, 34]. These physical and environmental challenges contribute to further reductions in physical activity and exacerbate declining functional reserves [35], encouraging transition to greater frailty severity. Remaining active during old age is important as sedentary lifestyle significantly increases risk of developing multiple chronic diseases and premature mortality [35]. Disease prevalence and functional impairment increases with age [36], which places the older adult at greater risk of frailty [5]. However, it is unclear whether age and age-related characteristics are associated with frailty in females with PD. This sample was randomly recruited from a population of females living independently in their own homes. Persons with PD seek institutional care sooner than non-PD counterparts [37], suggesting they may reach frailty sooner. Future longitudinal research may investigate onset of frailty in females with PD and follow frailty progression throughout the disease course. Also, further information on the contribution of physical activity to frailty onset and progression in PD is needed.

### 4.2. The Relationship between PD Management and Frailty

Accelerometers have been used in several research fields to monitor daily physical activity; monitoring of steps using waist-born accelerometers is feasible in PD populations and provides useful feedback on freezing (i.e., sudden inability to move, especially in the legs during walking), as well as long-term daily activity. Results of this study indicate that daily physical activity does not contribute to frailty phenotype in community-dwelling females with PD; however, daily carbidopa-levodopa dose was significantly related to frailty. Medication regimes for participants in this study included medications for the management of PD (i.e., carbidopa-levodopa, entacapone, pramipexole, amantadine, and ropinerole) and other conditions, such as anxiety/depression, blood pressure, migraine, muscle pain and inflammation, postmenopause symptoms, and difficulty sleeping. Multiple medication use in PD is associated with functional decline and high fall risk [38]; both of which are indicative of frailty. In addition, dopamine deficiency in PD may result in physical exhaustion [39], increasing ADL dependence risk for frailty. Therefore, the impact of PD medication regime on physical function and frailty requires further inquiry. 

In addition to motor impairments, persons with PD face secondary symptoms that impact basic daily function, such as depression, cognitive impairments, and nonmotor symptoms that increase functional dependence through increased anxiety, social isolation, and confusion [40]. Cognition is considered an important component of frailty [41] and is also associated with adverse PD outcomes [40]. Cognitive function can be influenced by depression and physical decline, and females with PD report greater incidence of both compared with males [14]. Also, females with PD report greater symptom-related stress and sleep disturbances than males [42, 43]. The importance of these non-motor symptoms cannot be underestimated, however they are beyond the scope of this study. Future research should consider how PD symptoms (motor and non-motor), disease severity, duration, other comorbidities, polypharmacy, and age impact frailty severity in both males and females with PD. 

### 4.3. Implications

#### 4.3.1. Community-Dwelling Populations and Neurological Disorders

The progressive nature of PD and related symptoms such as bradykinesia and tremor causes persons with PD to seek long-term care earlier than the general older adult population [37]. Females with PD may be more vulnerable to frailty than persons without neurological disorder because of PD-related systems that exacerbate the frailty phenotype. Therefore, it is important to make an early identification of frailty in community dwelling persons, especially those with neurological disorder who express a frailty phenotype earlier than the general population. 

#### 4.3.2. Frailty Management

Physical inactivity is directly linked to declining physiological reserve capacity, defined as adaptive responses (i.e., heart rate, blood pressure, respiration) that enable us to perform tasks or overcome external stresses [44]. In persons with PD the complexity of symptom presentation results in a reduced adaptive capacity. These losses of adaptive capacity, related to aging or disease, lead to declining functional independence and are a determinant of frailty [25]. Comorbidities and clinical symptoms interact between frailty and PD making identification of frailty in persons with PD challenging to diagnose [11]. Few studies have examined how to accurately identify frailty in this population [45, 46]. Early identification of frailty in persons with PD is relevant since over half of older adults living independently in their own homes are at-risk for frailty and subsequent functional decline [6]. Due to the transitional nature of both frailty and PD, the majority of disease progression occurs long before the individual requires institutional care [47]. As PD severity increases, symptoms become aggravated, further exacerbating underlying frailty [46]. Awareness of frailty during the initial stages of PD development may contribute to improved management strategies that delay and/or reverse frailty factors and preserve functional independence [6]. This study demonstrated that frailty was related to decreased physical activity in community-dwelling non-PD, and symptom management in PD females. Knowledge gained from the current study can be used to inform effective strategies for identifying prefrailty in non-PD females. This information can be applied to the development and delivery of timely support that addresses age- and disease-associated declines in function. Preventing functional decline has important implications for healthcare resource use in PD and non-PD older adults, as well as reducing physical, emotional, social, and financial problems attributed to frailty [25]. 

Currently, there is little evidence on how PD specifically impacts females and contributes to increased risk of frailty. Greater risk of frailty may be a consequence of greater functional declines [10], distress, and cognitive impairment [41], all resulting in ADL dependence [8]. In females with PD, physical activity participation should be aimed at managing PD symptoms. Managing PD symptoms, through physical activity and medication, will incidentally contribute to frailty management. Emphasis, therefore, should be placed on managing frailty within the context of PD symptoms [11]. Further collaboration is crucial between neurological, geriatric practice, and physical therapy/rehabilitation in terms of frailty assessment, progression, and addressing complications resulting in declining physical activity that may culminate in frailty.

## 5. Conclusion

Physical activity influences frailty expression in older females without PD, surprisingly no relationship between physical activity and frailty was found in our sample of females with PD. In PD, disease management may better indicate frailty severity. Further study is warranted to establish how PD-associated characteristics (i.e., polypharmacy) contribute to frailty and how physical activity participation interplays with the complex progress of frailty within PD. This study suggests PD-associated symptoms motivate community-dwelling females with PD to engage in leisure-time physical activities. Considering its vast impact in the community and on healthcare resources, identification of early frailty and management of resulting disability remains a priority area for geriatric research. Ultimately, enabling older adults to remain physically active promotes independence in ADL and empowers positive aging, albeit it may not protect females with PD from becoming frail.

## Figures and Tables

**Table 1:** Participant characteristics across frailty phenotypes in PD and non-PD females.

	PD			Non-PD		
	Nonfrail	Prefrail	Frail	Nonfrail	Prefrail	Frail
*N*	4	7	4	8	4	3
Age	69 ± 1	65 ± 10	63 ± 11	63 ± 8^ab^	79 ± 14^b^	90 ± 6
Body mass index (kg/m^2^)	26.31 ± 5.6	22.95 ± 4.3	25.06 ± 4.3	23.20 ± 5.7	34.60 ± 5.4	32.49 ± 12.2
Number of medications	4.0 ± 2.0	2.9 ± 1.5	3.3 ± 2.6	1.3 ± 1.3	6.0 ± 4.2	5.3 ± 0.6
mg carbidopa-levodopa per day	366.67 ± 57.7	571.43 ± 340.2	825.00 ± 330.4			
carbidopa-levodopa only (*N*)	2	3	2			
carbidopa-levodopa + pram (*N*)	2	2	0			
carbidopa-levodopa + enta (*N*)	0	0	1			
carbidopa-levodopa + enta + pram (*N*)	0	1	1			
carbidopa-levodopa + aman + rop (*N*)	0	1	0			
Hoehn & Yahr disease severity	1.83 ± 0.8	1.86 ± 0.6	2.50 ± 0.4			

PD: Parkinson's disease, *N*: number, kg: kilogram, m: meter, mg: milligram, pram: pramipexole, enta: entacapone, aman, amantadine, and rop: ropinirole.

^a^Significantly different from prefrail.

^b^Significantly different from frail.

**Table 2:** Main effects on physical activity variables.

	PD	Non-PD	Nonfrail	Prefrail	Frail
Number	15	15	12	11	7
Total steps	3476 ± 2814	3731 ± 3827	5624 ± 3309^a^	3019 ± 3290	1636 ± 1599
% time spent sedentary	61.7 ± 14.1	60.9 ± 16.6	49.39 ± 9.5^a^	66.4 ± 14.6	71.9 ± 11.3
% time at light activity	32.2 ± 10.6	31.7 ± 10.6	39.4 ± 7.4^a^	28.6 ± 10.2	25.2 ± 8.2
MLTA questionnaire	3052.3 ± 1611.6^b^	2015.0 ± 1517.4	3045.0 ± 1096.2^a^	2826.4 ± 1790.4^a^	1196.1 ± 1493.7

Analysis adjusted for age.

%: percent, MLTA: Minnesota Leisure Time Activity, and PD: Parkinson's disease.

^a^Significantly different from frail, *P* < 0.05.

^b^Significantly different from non-PD, *P* = 0.03.

**Table 3:** The relationship of physical activity variables to frailty severity in non-PD females.

	ANOVA	Spearman correlation		Linear regression	
	Main effect (*P*)	Correlation coefficient (*r*)	Significance (*P*)	Beta coefficient (*β*)	Significance (*P*)
Accelerometer: total counts					
Total steps counts^∗^	0.046	−0.79	0.001	1.113	0.202
Total activity counts	NS	−0.75	0.001		
Total activity time (min)	NS	−0.59	0.001		
Accelerometer: intensity					
% time spent sedentary^∗^	0.002	0.84	0.000	2.602	0.110
% time at light activity^∗^	0.012	−0.82	0.000	1.225	0.226
% time at MV activity	NS	−0.81	0.000		
GPS					
Total distance travelled (km)	NS	−0.63	0.012		
Average travel speed (km/h)	NS	−0.58	0.023		
Physical activity time (min)	NS	−0.54	0.036		
Self-reported activity					
MLTA questionnaire (kcal/week)^∗^	0.007	−0.82	0.000	−0.386	0.108

%: percentage; GPS: global positioning system; h: hour; kcal: kilocalories; km: kilometers; MLTA: Minnesota Leisure Time Activity; min: minutes; MV: moderate-vigorous; PD: Parkinson's disease; NS: nonsignificant (*P* > 0.05).

^∗^Significant main effects, therefore included in regression model.
